# Supplementary material for: Excess death estimates from multiverse analysis in 2009–2021
Source: medRxiv. 2022 Sep 23:2022.09.21.22280219. Preprint. [Version 1] doi: 10.1101/2022.09.21.22280219 (PMC9516863; doi:10.1101/2022.09.21.22280219)
Supplement: 1 [file NIHPP2022.09.21.22280219v1-supplement-1.pdf]

medRxiv preprint doi: https://doi.org/10.1101/2022.09.21.22280219; this version posted September 23, 2022. The copyright holder for this preprint (which was not certified by peer review) is the author/funder, who has granted medRxiv a license to display the preprint in perpetuity.

It is made available under a CC-BY 4.0 International license .

Supplementary Table and Figure Legends

Table S1: Distribution of the country rank of the excess death estimates in pandemic year 2021 expressed as a percentage of the expected deaths for the 33 countries as calculated for each of the 66 different reference baseline year sets. The countries are ordered by decreasing average rank (column 2); the standard deviation of the rank is also given in column 3. (A) Shows data for 2021 alone in Table S2A; (B) Shows summed data for 2019+2020+2021 in Table S2B; ; (C) Shows summed data for 2018+2019+2020+2021 in Table S2C. Such sums are labelled by their last year so all are marked as "2021". In most cases, the most common rank for a given year over the 66 reference years is on the diagonal.

Table S2: Variation with year from 2009 to 2021 of the average excess death expressed as a percentage of the expected death for all 33 countries studied here. The expected death is estimated from the average mortality values of each of the 66 different reference year sets, which are all combinations of one or more consecutive years from 2009 to 2019. The average over all the reference years is shown in each table. The four tables are (A) for the sum of two adjacent years recorded at the later year (the value for 2021 is 2020+2021), (B) for individual years, (C) for the sum of three adjacent years recorded at the later year (the value for 2021 is 2019+2020+2021) and (D) for the sum of four adjacent years recorded at the later year (the value for 2021 is 2018+2019+2020+2021). The shading is calibrated by range of values in all tables considered together. Each table is sorted by descending using value in the year column "2021".

medRxiv preprint doi: https://doi.org/10.1101/2022.09.21.22280219; this version posted September 23, 2022. The copyright holder for this preprint (which was not certified by peer review) is the author/funder, who has granted medRxiv a license to display the preprint in perpetuity.

It is made available under a CC-BY 4.0 International license .

Table S3: Comparing the percentage excess death in the pandemic years, 2020 and 2021, with that of other pairs of years between 2009 and 2019. In 9 of the 33 countries, the pandemic years, 2020+2021, marked as "2021" in red with yellow shading, were worse than any other pair of years.

Figure S1: Distribution of the year rank of the of the excess death expressed as a percentage of the expected death as calculated for each of the 66 different reference baseline year sets for 33 countries and for 12 pairs of years (2009+2010 to 2020+2021). The years are ordered by decreasing average rank (in column 2); the standard deviation of the rank is given in column 3. If the ranking for all 66 calculations were the same, all counts would be on the diagonal. This is true for 16 of the 33 countries. In the other 19 countries, the largest deviations from identical ranking occur for AUT (Austria) where the three years ranked in position 2 to 3 are 2011, 2013, and 2021. The mean rank for 2013 of 4.24 is not much less than that of 2021 of 4.52. Clearly the ranking of years for a given country are largely independent of the reference years used to calculate the expected deaths.

Figure S2 Variation with year from 2009 to 2021 of the excess death expressed as a percentage of the expected deaths as shown in Figure 1. The plots are for different projected periods (A) for individual years, (B) for the sum of three adjacent years (recorded at the latest year so the value for 2021 is 2019+2020+2021), and (C) for the sum of four adjacent years (recorded at the latest year so the value for 2021 is 2018+2019+2020+2021).

Table S1A: Country Rank Distribution of p%=X/E for Single Years

Location	LOC	Rank AVE	Rank SD	Rank in Sort from Highest p% to Lowest p% For Single Years																																
				1	2	3	4	5	6	7	8	9	10	11	12	13	14	15	16	17	18	19	20	21	22	23	24	25	26	27	28	29	30	31	32	33
Slovakia	SVK	1.26	0.47	50	15	1																														
Poland	POL	2.45	0.56		38	26	2																													
United States	USA	2.91	1.47	16	11	12	20	6			1																									
Latvia	LVA	3.55	0.56		2	26	38																													
Lithuania	LTU	5.20	0.94			1	6	48	5	3	2	1																								
Croatia	HRV	7.12	1.27					4	19	23	8	10	1	1																						
Czechia	CZE	7.23	1.17					4	20	5	32	4	1																							
Hungary	HUN	7.33	1.36					1	19	24	11	2	8	1																						
Estonia	EST	9.47	1.84					3		7	6	14	17	16	1	1			1																	
Greece	GRC	9.67	1.01						1		3	29	16	17																						
Chile	CHL	9.94	1.31						2	4	3	6	23	28																						
Portugal	PRT	12.56	1.18											1	46	9	6	2		1	1															
Austria	AUT	14.48	2.40												15	20	3	7	4	9	4	2	1													
United Kingdom	GBR	14.98	1.86												2	19	11	6	10	11	6															
Germany	DEU	15.35	2.03												1	6	22	13	10	5	3	2	1	1												
Italy	ITA	15.85	1.91											2	1	2	4	22	16	10	3	4	1													
Netherlands	NLD	16.70	1.37													2		10	17	18	15	2														
Slovenia	SVN	17.00	3.09													7	18	4	3	4	7	3	17	1												
Canada	CAN	19.45	1.77														2	2	2	3	4	30	14	2												
France	FRA	20.23	1.28																1	1	6	17	27	7												
Israel	ISR	22.06	1.35																1	1		2	3	30	26	1										
Spain	ESP	22.68	0.72																				2	24	34	5	1									
Finland	FIN	25.21	1.04																						1	13	34	10	5	3						
Belgium	BEL	25.52	1.22																							15	16	29	1	3	1					
Luxembourg	LUX	25.85	2.14																						5	23	5	10	6	5	4					
Switzerland	CHE	27.23	1.22																							1	5	6	29	20	1		1			
Denmark	DNK	29.00	1.92																									3	12	16	5	8	2	3	2	
Iceland	ISL	30.48	2.57																					1		1	1	1	3	6	12	11	4	15	1	3
New Zealand	NZL	30.62	1.48																									1	1	2	24	22	7	1	3	1
Australia	AUS	31.67	0.89																										1		1	17	41	5		
Norway	NOR	31.92	1.27																												14	8	9	34		
Sweden	SWE	33.95	0.61																												1			4	56	5
South Korea	KOR	34.77	0.62																														1	4	4	57

medRxiv preprint doi: https://doi.org/10.1101/2022.09.21.22280219; this version posted September 23, 2022. The copyright holder for this preprint (which was not certified by peer review) is the author/funder, who has granted medRxiv a license to display the preprint in perpetuity.
It is made available under a CC-BY 4.0 International license .

Table S1B: Country Rank Distribution of p%=X/E Summing Three Adjacent Years

Location	LOC	Rank AVE	Rank SD	Rank in Sort from Highest p% to Lowest p% Summing Three Adjacent Years																																
				1	2	3	4	5	6	7	8	9	10	11	12	13	14	15	16	17	18	19	20	21	22	23	24	25	26	27	28	29	30	31	32	33
United States	USA	1.27	0.54	50	15		1																													
Poland	POL	1.79	0.44	15	50	1																														
Slovakia	SVK	4.94	3.56	1	1	39	7	1	3	2	1	1	3	3			2	1			1															
Czechia	CZE	5.58	2.15				34	8	6	4	6	5	1		1	1																				
Greece	GRC	6.00	2.95			11	8	20	7	8	3	2	1	1	2		2				1															
Hungary	HUN	6.95	1.66				7	5	10	23	10	7	2	2																						
Lithuania	LTU	7.02	2.33			5	2	10	16	4	14	6	4	2	1	2																				
Latvia	LVA	7.85	2.69			2		8	11	12	13	6	6	4	1	1		1				1														
Croatia	HRV	8.76	2.47			2	3	3		8	7	23	11	2	2	3	1			1																
Italy	ITA	9.77	2.94			4			8	2	1	8	16	12	8	2	2	1	1		1															
Chile	CHL	11.33	4.68			2	1	7	4		6	1	7	8	8	1	3	5	4	1	2	3	2													
United Kingdom	GBR	12.52	3.61				3	2	1	1	4	1	2	9	7	4	9	6	13	2	1															
Portugal	PRT	13.47	2.40					1				2	2	3	8	21	12	9	3	3					1											
Slovenia	SVN	14.24	3.80								1	2	4	11	15	2	5	8			4	5	7													
France	FRA	14.98	2.98							1		1	1	3	6	9	7	14	8	4	4	4	3	1												
Austria	AUT	16.92	3.33										1	2		10	8	6	7	2	4	8	15	1												
Spain	ESP	17.02	3.64					1				1	2	1	2	2	5	2	8	16	7	4	3	3	3	2										
Netherlands	NLD	17.91	2.71										1	1		1	1	7	9	11	5	9	3	3	2		1									
Canada	CAN	17.95	3.21										2		2	4	2	3	7	9	6	11	5	8	1	1										
Germany	DEU	20.38	2.45												1				4	2	5	9	18	5	5	3	4									
Estonia	EST	20.71	5.10							1				2	2	3	7	1		3			4	9	14	5	7	3	1	1	2					
Belgium	BEL	21.76	1.64																1		5		4	35	14	3	1									
Israel	ISR	23.83	1.55															1						1	17	29	13	3	1							
Switzerland	CHE	24.80	1.17																			1			7	14	26	17		1						
Luxembourg	LUX	25.64	1.73																			1	1		1	9	7	33	6	5						
Finland	FIN	27.53	0.82																									2	38	16	1					
Iceland	ISL	29.06	3.03																			1			1		2	7	12	12	6	5	6	2	6	2
Denmark	DNK	29.92	2.05																										1	24	4	10	7	3	6	
New Zealand	NZL	30.38	1.73																									1	2	1	26	11	5	2	2	3
Sweden	SWE	30.79	1.47																								1		1	2	8	19	24	2		
Australia	AUS	32.95	1.16																										1			1	11	33	18	1
Norway	NOR	33.00	1.14																												1	10	7	18	30	
South Korea	KOR	34.83	0.69																												1			1	4	60

medRxiv preprint doi: https://doi.org/10.1101/2022.09.21.22280219; this version posted September 23, 2022. The copyright holder for this preprint (which was not certified by peer review) is the author/funder, who has granted medRxiv a license to display the preprint in perpetuity.
It is made available under a CC-BY 4.0 International license .

Table S1C: Country Rank Distribution of p%=X/E Summing Three Adjacent Years

	Rank	Rank	Rank in Sort from Highest p% to Lowest p%																																		
LOC	AVE	SD	1	2	3	4	5	6	7	8	9	10	11	12	13	14	15	16	17	18	19	20	21	22	23	24	25	26	27	28	29	30	31	32	33	34	35
USA	1.30	0.55	48	17		1																															
POL	1.79	0.48	16	48	2																																
HUN	6.11	1.93			5	9	12	17	8	7	4	2	2																								
LVA	6.76	3.27			5	6	25	2	9	4	6	1	2	1	2		1		1		1																
CZE	6.97	4.03			6	23	7	3	7	2	2	3	1	2		5	1	4																			
SVK	7.12	5.74	2		25	11	2	2	3	3	1	1	2		2	1		1	3	3	1	2	1														
GRC	7.39	3.79			6	5	9	14	11	6	3	2	1	2	1	2	1		1		1			1													
LTU	9.33	3.42			2	2	4	5	8	6	12	4	8	4	4	2	2	1		1		1															
HRV	9.35	3.69			4	2		3	3	23	8	8	3	2	1	1	1	2	3		1		1														
ITA	10.44	3.84			2	2		6	6	2	9	12	4	3	7	6	1	2	1	1			1	1													
GBR	11.21	4.93			8	2	2	5	1		4	3	8	3	3	6	4	9	4	2	1		1														
PRT	12.18	2.93				1			2	2	5	5	11	14	11	2	5	4	1	2					1												
FRA	14.00	4.08					1	1	2	1	2	2	6	8	15	3	8	1	3	2	3	1	2	4		1											
CHL	15.17	6.07		1	1		3	6	1	1	1	3	3	3	3	2	1	2		3	10	5	13	2	1	1											
SVN	15.45	4.27								3		7	7	7	3	3	2	2	4	4	10	5	6	3													
CAN	16.09	4.62				1	1		2	2	1	3	2	1	1	5	7	6	9	5	2	4	7	4	2	1											
DEU	16.12	4.35							1	1	3	3	1	3	1	9	11	8	6	2	1	5	3	1	2	1	3	1									
NLD	16.41	3.48								2	2		1	4		7	8	11	7	7	7	3	2	1	3		1										
ESP	16.62	4.47				1		1	2			4	1	1	3	5	3	7	10	8	2	6	2	3	4	3											
AUT	17.56	3.75								1	1	2	2	5	2	3	3	1	4	3	7	18	12	2													
EUM	18.21	1.76													2	1	3	3	8	17	16	13	3														
EST	21.17	5.75						1			2	1	1	2	4	3	1	1		1	1	1		10	14	6	5	3	4	1	1	1	2				
BEL	21.65	1.81															1		1	5	2	1	7	31	14	3		1									
ISR	24.21	1.67															1						2		15	15	25	6	1	1							
LUX	24.83	2.84													1		1	1					2	2	3	18	5	17	9	5	2						
CHE	25.08	1.35																				1			6	12	21	22	1	2	1						
FIN	27.48	0.82																									1	2	36	18	9						
ISL	28.97	3.85												1										1	1	2	3	8	7	8	7	4	5	6	4	6	3
DNK	29.47	1.99																										2	3	25	10	4	12	5		5	
TWN	29.89	2.22																								3	1	3	2	2	15	7	19	8	6		
NZL	30.17	1.77																									1	1	1	2	14	30	6	6		2	3
SWE	30.74	1.76																					1						1	2	7	14	11	29	1		
NOR	32.58	1.13																														4	10	8	32	12	
AUS	33.36	1.14																											1			1	1	4	21	37	1
KOR	34.80	0.72																														1			2	4	59

medRxiv preprint doi: https://doi.org/10.1101/2022.09.21.22280219; this version posted September 23, 2022. The copyright holder for this preprint (which was not certified by peer review) is the author/funder, who has granted medRxiv a license to display the preprint in perpetuity.
It is made available under a CC-BY 4.0 International license .

Table S2A: Relative Excess Death (p%=X/E) Summing Two Adjacent Years for All Countries and All Years

Location	LOC	2009	2010	2011	2012	2013	2014	2015	2016	2017	2018	2019	2020	2021
United States	USA	NA	1.34	0.97	0.53	-0.04	-0.64	-0.38	-0.04	0.03	-0.19	-1.25	7.10	16.69
Poland	POL	NA	7.83	4.62	3.11	2.21	-1.06	-2.19	-2.88	-3.79	-2.47	-3.26	3.01	14.16
Slovakia	SVK	NA	9.09	6.64	3.90	2.39	-0.48	-0.95	-2.23	-4.27	-4.55	-6.86	-4.47	10.22
Czechia	CZE	NA	8.25	5.73	3.95	2.89	-0.14	-1.20	-2.41	-4.54	-4.23	-5.64	-0.59	8.65
Lithuania	LTU	NA	6.44	4.70	2.18	1.78	0.12	-0.36	-0.04	-2.52	-4.57	-7.02	-2.48	8.48
Croatia	HRV	NA	7.89	4.94	2.69	0.29	-2.08	-0.01	-0.89	-2.88	-2.90	-5.52	-2.54	6.86
Latvia	LVA	NA	7.74	4.97	2.35	1.42	-0.56	-1.96	-2.85	-2.99	-2.77	-4.50	-4.53	6.81
Chile	CHL	NA	6.50	5.67	3.45	3.13	2.03	1.08	-1.67	-3.94	-5.50	-6.93	-1.78	6.74
Hungary	HUN	NA	6.13	4.43	2.81	0.99	-1.21	-0.64	-1.82	-2.98	-2.33	-4.35	-2.03	6.70
Greece	GRC	NA	5.65	3.83	4.11	1.56	-2.17	-1.34	-1.89	-1.97	-2.46	-3.28	-0.44	5.64
Italy	ITA	NA	4.61	3.34	3.63	1.55	-1.70	-0.24	-0.84	-2.05	-2.21	-4.49	2.09	5.47
Slovenia	SVN	NA	7.72	4.89	4.02	3.07	-0.39	-1.70	-2.41	-3.27	-3.50	-5.49	0.63	4.69
United Kingdom	GBR	NA	4.83	2.27	0.90	0.97	-0.73	-0.27	-0.09	-1.46	-1.64	-3.66	0.46	4.19
Spain	ESP	NA	4.86	2.56	2.61	0.88	-1.77	0.14	-0.08	-1.61	-1.64	-4.54	1.14	3.60
Portugal	PRT	NA	6.65	3.47	1.84	1.80	-1.03	-1.97	-0.98	-2.09	-2.75	-3.44	-0.51	3.52
Austria	AUT	NA	5.84	3.44	3.09	3.35	0.78	-0.35	-2.13	-4.04	-3.59	-4.51	-0.79	3.31
Netherlands	NLD	NA	4.86	2.99	2.07	1.55	-1.18	-1.43	-0.43	-1.44	-2.00	-3.48	-0.80	2.51
France	FRA	NA	5.74	2.75	1.77	1.63	-1.56	-1.73	-0.85	-1.55	-1.89	-2.90	0.29	2.29
Canada	CAN	NA	5.36	2.78	1.23	0.18	0.24	-0.26	-1.67	-1.60	-1.42	-3.08	-0.39	2.27
Estonia	EST	NA	12.57	8.10	4.59	2.18	0.50	-1.89	-4.58	-5.37	-5.61	-7.08	-7.68	1.46
Belgium	BEL	NA	6.27	3.67	3.18	3.69	0.01	-1.37	-1.57	-3.32	-3.49	-5.26	0.40	1.42
Germany	DEU	NA	4.76	2.76	1.24	1.54	-0.66	-1.35	-1.35	-2.61	-1.27	-1.90	-1.70	1.00
Switzerland	CHE	NA	6.84	4.05	2.69	2.81	0.58	0.40	-1.00	-3.66	-4.35	-5.95	-1.71	-1.19
Israel	ISR	NA	5.61	4.88	4.31	2.05	-0.52	-0.07	-0.82	-2.93	-4.50	-5.10	-3.22	-1.34
Luxembourg	LUX	NA	8.65	8.06	5.97	2.64	-1.32	-2.93	-4.85	-4.36	-2.70	-4.77	-3.44	-2.74
Finland	FIN	NA	8.01	6.05	3.88	2.03	-0.13	-1.65	-2.38	-3.35	-4.17	-5.42	-6.50	-5.39
Sweden	SWE	NA	6.09	4.69	4.16	3.35	0.57	-0.70	-1.75	-3.18	-4.12	-7.59	-6.31	-6.63
Iceland	ISL	NA	5.32	3.01	-1.56	0.33	0.53	-1.43	1.80	0.85	-2.87	-4.59	-6.04	-7.25
Denmark	DNK	NA	11.73	7.48	3.39	1.34	-1.35	-2.99	-3.42	-4.33	-3.95	-5.31	-8.14	-7.79
New Zealand	NZL	NA	5.30	4.63	4.51	0.43	-0.76	0.04	-2.66	-2.69	-2.38	-3.93	-7.26	-8.98
Norway	NOR	NA	7.24	5.83	4.96	3.44	0.38	-1.45	-2.67	-3.97	-5.09	-6.87	-9.06	-9.41
Australia	AUS	NA	6.98	5.69	4.70	2.19	0.75	-0.11	-2.16	-2.53	-4.63	-7.04	-8.84	-9.49
South Korea	KOR	NA	13.61	10.18	8.04	5.26	0.90	-1.73	-3.60	-6.04	-7.33	-9.81	-12.65	-13.12

medRxiv preprint doi: https://doi.org/10.1101/2022.09.21.22280219; this version posted September 23, 2022. The copyright holder for this preprint (which was not certified by peer review) is the author/funder, who has granted medRxiv a license to display the preprint in perpetuity.
It is made available under a CC-BY 4.0 International license .

Table S2B: Percentage Excess Death (p%=X/E) in a Single Year for All Countries and All Years

Location	LOC	2009	2010	2011	2012	2013	2014	2015	2016	2017	2018	2019	2020	2021
Slovakia	SVK	9.31	8.98	4.43	3.61	1.30	-2.12	0.29	-4.59	-4.02	-4.98	-8.61	-0.32	20.69
Poland	POL	9.39	5.92	2.97	2.88	1.20	-3.60	-1.15	-4.90	-3.02	-2.25	-4.57	10.18	17.77
United States	USA	1.75	0.97	1.00	0.08	-0.13	-1.11	0.37	-0.43	0.50	-0.85	-1.62	15.80	17.60
Latvia	LVA	7.55	7.85	2.03	2.60	0.17	-1.36	-2.63	-3.15	-2.91	-2.70	-6.37	-2.75	16.31
Lithuania	LTU	6.52	6.63	3.05	1.59	2.23	-1.71	1.24	-1.06	-3.72	-5.17	-8.61	3.89	13.33
Hungary	HUN	6.57	5.67	3.18	2.43	-0.46	-1.97	0.67	-4.29	-1.70	-2.97	-5.71	1.59	11.80
Croatia	HRV	8.77	6.97	2.89	2.43	-1.89	-2.33	2.23	-4.02	-1.80	-4.04	-7.03	1.86	11.80
Czechia	CZE	9.58	6.84	4.54	3.27	2.41	-2.73	0.21	-5.07	-4.12	-4.43	-6.92	5.55	11.62
Chile	CHL	4.67	8.70	3.15	4.18	2.53	1.95	0.63	-3.45	-4.04	-6.57	-6.94	3.58	10.15
Greece	GRC	6.66	4.32	3.01	4.84	-1.98	-2.68	-0.36	-3.71	-0.55	-4.64	-2.24	1.04	9.91
Estonia	EST	14.10	10.72	5.17	3.68	0.38	0.30	-4.35	-5.12	-5.93	-5.61	-8.83	-6.84	9.41
Portugal	PRT	6.97	6.22	0.67	2.87	0.63	-2.77	-1.28	-0.79	-3.46	-2.15	-4.80	3.61	3.33
Austria	AUT	6.89	4.77	2.09	4.02	2.65	-1.09	0.32	-4.57	-3.55	-3.68	-5.38	3.73	2.85
United Kingdom	GBR	5.65	4.11	0.54	1.33	0.69	-2.06	1.59	-1.63	-1.22	-1.98	-5.26	6.16	2.30
Italy	ITA	6.15	3.12	3.54	3.71	-0.57	-2.82	2.27	-3.91	-0.23	-4.18	-4.80	8.90	2.07
Germany	DEU	5.31	4.08	1.33	1.02	1.94	-3.30	0.45	-3.21	-2.13	-0.52	-3.36	-0.17	2.06
Slovenia	SVN	9.41	5.96	3.89	4.03	2.02	-2.84	-0.70	-4.17	-2.49	-4.59	-6.47	7.51	1.82
Netherlands	NLD	5.37	4.43	1.67	2.54	0.65	-2.89	0.06	-0.84	-1.96	-1.97	-4.90	3.28	1.81
Canada	CAN	7.23	3.47	1.98	0.38	-0.13	0.48	-1.13	-2.29	-1.03	-1.90	-4.33	3.47	1.01
France	FRA	6.58	4.70	0.63	2.67	0.41	-3.68	-0.03	-1.85	-1.45	-2.52	-3.47	3.81	0.61
Israel	ISR	6.15	5.08	4.67	4.09	0.05	-1.09	0.90	-2.51	-3.35	-5.64	-4.59	-1.90	-0.82
Spain	ESP	6.67	3.24	2.02	3.32	-1.37	-2.04	2.40	-2.40	-0.73	-2.41	-6.51	8.78	-1.44
Finland	FIN	8.13	7.94	4.26	3.56	0.59	-0.79	-2.46	-2.26	-4.38	-3.93	-6.85	-6.12	-4.63
Belgium	BEL	7.25	5.39	2.07	4.34	3.12	-2.97	0.29	-3.32	-3.25	-3.66	-6.76	7.57	-4.67
Luxembourg	LUX	8.05	9.29	6.93	5.10	0.31	-2.84	-3.07	-6.52	-2.20	-3.15	-6.31	-0.57	-4.78
Switzerland	CHE	7.85	5.88	2.29	3.10	2.55	-1.32	2.11	-4.01	-3.30	-5.35	-6.51	3.01	-5.33
Denmark	DNK	12.91	10.22	4.44	2.02	0.34	-3.33	-2.98	-4.17	-4.94	-3.28	-7.60	-8.95	-6.93
Iceland	ISL	5.39	5.36	0.83	-3.77	4.45	-3.20	0.40	3.26	-1.41	-4.19	-5.04	-6.93	-7.48
New Zealand	NZL	7.50	2.99	6.03	2.85	-2.11	0.37	-0.45	-4.97	-0.64	-4.25	-3.89	-10.68	-7.48
Australia	AUS	8.17	5.97	5.53	4.00	0.53	1.06	-1.12	-3.07	-1.92	-7.17	-6.83	-10.70	-8.22
Norway	NOR	7.74	6.87	4.94	5.11	1.92	-1.01	-1.76	-3.44	-4.37	-5.69	-7.91	-10.07	-8.65
Sweden	SWE	6.66	6.01	3.86	4.93	2.26	-0.64	-0.32	-2.72	-3.19	-4.61	-10.10	-2.16	-10.61
South Korea	KOR	15.53	12.16	8.59	7.82	3.10	-0.92	-2.25	-4.65	-7.11	-7.31	-11.99	-13.07	-12.96

medRxiv preprint doi: https://doi.org/10.1101/2022.09.21.22280219; this version posted September 23, 2022. The copyright holder for this preprint (which was not certified by peer review) is the author/funder, who has granted medRxiv a license to display the preprint in perpetuity.
It is made available under a CC-BY 4.0 International license .

Table S2C: Relative Excess Death (p%=X/E) Summing Three Adjacent Years for All Countries and All Years

Location	LOC	2009	2010	2011	2012	2013	2014	2015	2016	2017	2018	2019	2020	2021
United States	USA	NA	NA	1.21	0.66	0.29	-0.41	-0.30	-0.40	0.13	-0.28	-0.69	4.47	10.61
Poland	POL	NA	NA	6.45	4.29	2.72	0.48	-0.84	-2.87	-2.69	-3.03	-2.95	1.51	8.22
Slovakia	SVK	NA	NA	7.59	5.69	3.13	0.93	-0.15	-2.12	-2.77	-4.50	-5.87	-4.58	4.12
Czechia	CZE	NA	NA	7.09	4.99	3.52	1.07	0.07	-2.43	-2.91	-4.42	-5.06	-1.76	3.63
Greece	GRC	NA	NA	4.84	4.28	2.12	0.20	-1.46	-2.06	-1.35	-2.79	-2.30	-1.74	3.13
Lithuania	LTU	NA	NA	5.24	3.59	2.14	0.54	0.43	-0.66	-1.33	-3.46	-5.98	-3.43	2.72
Hungary	HUN	NA	NA	5.23	3.85	1.80	0.08	-0.48	-1.78	-1.69	-2.89	-3.39	-2.26	2.68
Latvia	LVA	NA	NA	6.04	4.38	1.83	0.69	-1.06	-2.16	-2.67	-2.65	-3.77	-3.72	2.62
Croatia	HRV	NA	NA	6.32	4.21	1.25	-0.49	-0.52	-1.26	-1.09	-3.17	-4.19	-2.93	2.32
Chile	CHL	NA	NA	5.18	4.99	2.98	2.57	1.40	-0.65	-2.64	-4.98	-6.13	-3.46	2.17
Italy	ITA	NA	NA	4.18	3.41	2.14	0.01	-0.40	-1.54	-0.69	-2.82	-3.14	-0.02	2.03
United Kingdom	GBR	NA	NA	3.40	1.99	0.87	-0.02	0.08	-0.70	-0.44	-1.60	-2.83	-0.31	1.11
Slovenia	SVN	NA	NA	6.46	4.70	3.38	1.09	-0.45	-2.50	-2.39	-3.68	-4.47	-1.04	1.07
Portugal	PRT	NA	NA	4.79	3.45	1.60	0.42	-0.95	-1.40	-1.66	-1.95	-3.29	-0.89	0.94
France	FRA	NA	NA	4.15	2.88	1.45	-0.03	-0.89	-1.63	-0.92	-1.75	-2.29	-0.50	0.53
Austria	AUT	NA	NA	4.61	3.68	2.97	1.88	0.66	-1.76	-2.58	-3.88	-4.16	-1.71	0.46
Spain	ESP	NA	NA	3.85	2.78	1.22	-0.15	-0.39	-0.76	-0.34	-1.92	-3.33	-0.06	0.24
Canada	CAN	NA	NA	4.28	2.04	0.85	0.37	-0.14	-0.88	-1.37	-1.63	-2.34	-0.81	0.16
Netherlands	NLD	NA	NA	3.81	2.88	1.63	0.08	-0.71	-1.19	-0.91	-1.58	-2.95	-1.14	0.13
Germany	DEU	NA	NA	3.77	2.34	1.65	0.06	-0.12	-1.82	-1.45	-1.75	-1.82	-1.16	-0.28
Belgium	BEL	NA	NA	4.84	3.91	3.17	1.43	0.11	-2.02	-2.13	-3.43	-4.59	-0.93	-1.28
Estonia	EST	NA	NA	10.37	6.89	3.44	1.82	-0.88	-2.72	-4.77	-5.19	-6.44	-6.74	-1.69
Israel	ISR	NA	NA	5.21	4.53	2.82	0.90	-0.10	-0.97	-1.75	-3.91	-4.59	-4.07	-2.45
Switzerland	CHE	NA	NA	5.25	3.70	2.61	1.37	1.07	-1.13	-1.81	-4.26	-5.11	-2.92	-2.96
Luxembourg	LUX	NA	NA	8.11	7.24	4.20	0.92	-1.76	-4.04	-3.78	-3.79	-3.78	-3.20	-3.77
Finland	FIN	NA	NA	6.83	5.30	2.85	1.16	-0.84	-1.77	-2.97	-3.46	-5.00	-5.58	-5.78
Iceland	ISL	NA	NA	3.77	0.68	0.47	-0.89	0.47	0.15	0.67	-0.89	-3.63	-5.46	-6.55
New Zealand	NZL	NA	NA	5.56	4.03	2.26	0.41	-0.65	-1.67	-1.96	-3.21	-2.89	-6.27	-7.33
Denmark	DNK	NA	NA	9.65	6.01	2.73	0.13	-1.54	-3.03	-3.58	-3.67	-4.85	-6.22	-7.40
Sweden	SWE	NA	NA	5.18	4.61	3.37	1.85	0.12	-1.54	-2.39	-3.81	-6.29	-5.89	-7.90
Australia	AUS	NA	NA	6.31	4.95	3.11	1.65	-0.05	-1.27	-2.21	-4.25	-5.51	-8.42	-8.75
Norway	NOR	NA	NA	6.42	5.55	3.90	1.91	-0.37	-2.15	-3.27	-4.58	-6.08	-7.99	-8.95
South Korea	KOR	NA	NA	11.78	9.30	6.26	3.05	-0.25	-2.80	-4.88	-6.53	-9.00	-10.99	-12.80

medRxiv preprint doi: https://doi.org/10.1101/2022.09.21.22280219; this version posted September 23, 2022. The copyright holder for this preprint (which was not certified by peer review) is the author/funder, who has granted medRxiv a license to display the preprint in perpetuity.
It is made available under a CC-BY 4.0 International license .

Table S2D: Relative Excess Death (p%=X/E) Summing Four Adjacent Years for All Countries and All Years

Location	LOC	2009	2010	2011	2012	2013	2014	2015	2016	2017	2018	2019	2020	2021
Australia	AUS	NA	NA	NA	5.65	3.75	2.53	0.87	-0.88	-1.48	-3.54	-4.96	-6.90	-8.41
Austria	AUT	NA	NA	NA	4.43	3.39	1.91	1.45	-0.72	-2.24	-2.88	-4.29	-2.18	-0.58
Belgium	BEL	NA	NA	NA	4.67	3.67	1.55	1.10	-0.81	-2.37	-2.56	-4.32	-1.53	-1.90
Canada	CAN	NA	NA	NA	3.37	1.58	0.85	0.09	-0.60	-0.82	-1.41	-2.24	-0.77	-0.26
Switzerland	CHE	NA	NA	NA	4.62	3.33	1.52	1.49	-0.31	-1.76	-2.79	-4.91	-3.08	-3.60
Chile	CHL	NA	NA	NA	4.70	4.15	2.51	1.87	-0.07	-1.73	-3.85	-5.67	-3.76	-0.08
Czechia	CZE	NA	NA	NA	6.19	4.41	2.00	0.93	-1.17	-2.78	-3.22	-4.98	-2.26	1.74
Germany	DEU	NA	NA	NA	3.20	2.37	0.49	0.28	-0.79	-1.78	-1.10	-2.04	-1.28	-0.23
Denmark	DNK	NA	NA	NA	8.02	4.88	1.49	-0.36	-1.91	-3.22	-3.21	-4.40	-5.63	-6.13
Spain	ESP	NA	NA	NA	3.71	1.71	0.38	0.51	-0.91	-0.75	-0.87	-3.11	-0.22	-0.41
Estonia	EST	NA	NA	NA	8.87	5.43	2.84	0.44	-1.76	-3.34	-4.79	-5.92	-6.35	-2.46
Finland	FIN	NA	NA	NA	6.05	4.14	1.97	0.28	-1.14	-2.38	-3.16	-4.27	-5.23	-5.28
France	FRA	NA	NA	NA	3.88	2.35	0.23	0.07	-1.04	-1.49	-1.23	-2.09	-0.64	-0.13
United Kingdom	GBR	NA	NA	NA	2.87	1.66	0.12	0.39	-0.35	-0.83	-0.83	-2.53	-0.53	0.36
Greece	GRC	NA	NA	NA	5.01	2.81	1.03	0.21	-1.89	-1.53	-2.05	-2.50	-1.31	1.36
Croatia	HRV	NA	NA	NA	5.46	2.78	0.46	0.33	-1.28	-1.27	-1.72	-4.03	-2.54	0.89
Hungary	HUN	NA	NA	NA	4.65	2.88	0.97	0.36	-1.33	-1.61	-1.90	-3.49	-2.00	1.40
Iceland	ISL	NA	NA	NA	1.77	1.60	-0.53	-0.61	1.14	-0.30	-0.63	-2.01	-4.53	-6.02
Israel	ISR	NA	NA	NA	4.87	3.31	1.75	0.86	-0.77	-1.63	-2.79	-4.12	-3.93	-3.25
Italy	ITA	NA	NA	NA	4.10	2.42	0.91	0.64	-1.26	-1.16	-1.54	-3.28	-0.03	0.55
South Korea	KOR	NA	NA	NA	10.64	7.57	4.27	1.57	-1.49	-4.01	-5.59	-8.04	-10.14	-11.57
Lithuania	LTU	NA	NA	NA	4.23	3.16	1.07	0.63	-0.03	-1.51	-2.38	-4.84	-3.58	0.68
Luxembourg	LUX	NA	NA	NA	7.71	5.69	2.61	0.12	-2.76	-3.32	-3.38	-4.21	-2.66	-3.40
Latvia	LVA	NA	NA	NA	5.34	3.49	1.19	0.01	-1.42	-2.19	-2.52	-3.45	-3.36	1.45
Netherlands	NLD	NA	NA	NA	3.51	2.33	0.48	0.10	-0.72	-1.37	-1.16	-2.42	-1.32	-0.36
Norway	NOR	NA	NA	NA	6.06	4.60	2.63	0.95	-1.18	-2.74	-3.91	-5.46	-7.13	-8.18
New Zealand	NZL	NA	NA	NA	5.02	2.60	1.92	0.34	-1.63	-1.26	-2.41	-3.25	-4.78	-6.46
Poland	POL	NA	NA	NA	5.77	3.73	1.31	0.28	-1.67	-2.69	-2.36	-3.22	0.61	5.86
Portugal	PRT	NA	NA	NA	4.37	2.79	0.55	0.05	-0.84	-1.87	-1.72	-2.62	-1.46	0.24
Slovakia	SVK	NA	NA	NA	6.64	4.63	1.85	0.83	-1.23	-2.55	-3.28	-5.50	-4.38	1.96
Slovenia	SVN	NA	NA	NA	5.91	4.08	1.84	0.70	-1.34	-2.43	-2.89	-4.33	-1.32	-0.24
Sweden	SWE	NA	NA	NA	4.98	3.88	2.21	1.17	-0.74	-2.09	-3.08	-5.55	-5.36	-7.22
United States	USA	NA	NA	NA	0.96	0.50	-0.03	-0.17	-0.30	-0.14	-0.09	-0.59	3.54	7.82

medRxiv preprint doi: https://doi.org/10.1101/2022.09.21.22280219; this version posted September 23, 2022. The copyright holder for this preprint (which was not certified by peer review) is the author/funder, who has granted medRxiv a license to display the preprint in perpetuity.
It is made available under a CC-BY 4.0 International license .

Table S3: Three Worst Pairs of Years, Plus 2021 If Not Already Included

Location	LOC	Year1	Year2	Year3	Year4	p1%	p2%	p3%	p4%	pd1%	pd2%	pd3%	pd4%
Australia	AUS	2010	2011	2012	2021	6.6	5.4	4.4	-9.7	16.4	15.1	14.1	0.00
Austria	AUT	2010	2011	2013	2021	5.7	3.3	3.2	3.2	2.5	0.1	0.0	0.00
Belgium	BEL	2010	2013	2011	2021	6.3	3.7	3.7	1.4	4.9	2.3	2.3	0.00
Canada	CAN	2010	2011	2021	NA	5.3	2.7	2.2		3.1	0.5	0.0	
Switzerland	CHE	2010	2011	2013	2021	6.7	3.9	2.7	-1.3	8.0	5.2	4.0	0.00
Chile	CHL	2021	2010	2011	NA	6.5	6.1	5.3		1.2	0.8	0.0	
Czechia	CZE	2021	2010	2011	NA	8.7	8.3	5.8		2.9	2.5	0.0	
Germany	DEU	2010	2011	2013	2021	4.8	2.8	1.5	1.0	3.8	1.8	0.5	0.00
Denmark	DNK	2010	2011	2012	2021	12.0	7.7	3.6	-7.6	19.6	15.3	11.2	0.00
Spain	ESP	2010	2021	2012	NA	4.8	3.6	2.6		2.3	1.0	0.0	
Estonia	EST	2010	2011	2012	2021	12.8	8.3	4.8	1.7	11.2	6.7	3.2	0.00
Finland	FIN	2010	2011	2012	2021	8.1	6.1	4.0	-5.3	13.4	11.5	9.3	0.00
France	FRA	2010	2011	2021	NA	5.9	2.9	2.4		3.5	0.5	0.0	
United Kingdom	GBR	2010	2021	2011	NA	4.8	4.2	2.2		2.6	1.9	0.0	
Greece	GRC	2021	2010	2012	NA	5.6	5.6	4.0		1.6	1.5	0.0	
Croatia	HRV	2010	2021	2011	NA	8.1	7.0	5.1		3.0	1.9	0.0	
Hungary	HUN	2021	2010	2011	NA	6.8	6.3	4.6		2.3	1.7	0.0	
Iceland	ISL	2010	2011	2016	2021	5.2	2.9	1.7	-7.3	12.5	10.2	9.0	0.00
Israel	ISR	2010	2011	2012	2021	5.4	4.6	4.1	-1.5	6.9	6.2	5.7	0.00
Italy	ITA	2021	2010	2012	NA	5.4	4.6	3.6		1.8	1.0	0.0	
South Korea	KOR	2010	2011	2012	2021	13.1	9.7	7.5	-13.5	26.5	23.1	21.0	0.00
Lithuania	LTU	2021	2010	2011	NA	8.6	6.6	4.8		3.8	1.7	0.0	
Luxembourg	LUX	2010	2011	2012	2021	8.8	8.2	6.1	-2.6	11.4	10.8	8.7	0.00
Latvia	LVA	2010	2021	2011	NA	8.0	7.0	5.2		2.8	1.8	0.0	
Netherlands	NLD	2010	2011	2021	NA	4.9	3.0	2.5		2.4	0.5	0.0	
Norway	NOR	2010	2011	2012	2021	7.2	5.8	4.9	-9.4	16.6	15.2	14.4	0.00
New Zealand	NZL	2010	2011	2012	2021	5.2	4.5	4.4	-9.1	14.2	13.6	13.5	0.00
Poland	POL	2021	2010	2011	NA	14.2	7.9	4.7		9.5	3.2	0.0	
Portugal	PRT	2010	2021	2011	NA	6.8	3.6	3.6		3.2	0.0	0.0	
Slovakia	SVK	2021	2010	2011	NA	10.2	9.1	6.6		3.6	2.5	0.0	
Slovenia	SVN	2010	2011	2021	NA	7.7	5.0	4.7		3.0	0.3	0.0	
Sweden	SWE	2010	2011	2012	2021	6.0	4.6	4.1	-6.7	12.7	11.3	10.8	0.00
United States	USA	2021	2010	2011	NA	16.6	1.3	0.9		15.7	0.4	0.0	

medRxiv preprint doi: https://doi.org/10.1101/2022.09.21.22280219; this version posted September 23, 2022. The copyright holder for this preprint (which was not certified by peer review) is the author/funder, who has granted medRxiv a license to display the preprint in perpetuity.
It is made available under a CC-BY 4.0 International license .

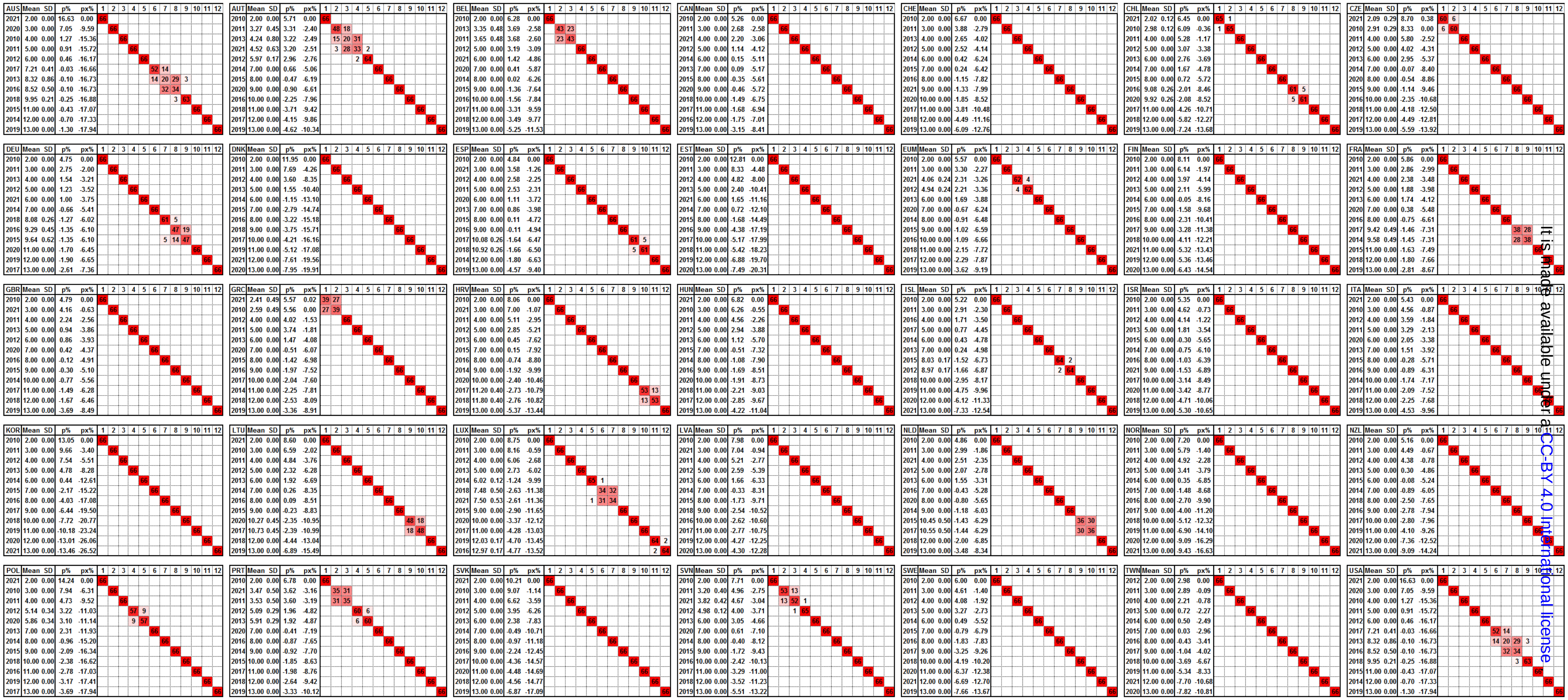

Figure S1

medRxiv preprint doi: https://doi.org/10.1101/2022.09.21.22280219; this version posted September 23, 2022. The copyright holder for this preprint (which was not certified by peer review) is the author/funder, who has granted medRxiv a license to display the preprint in perpetuity. It is made available under a CC-BY 4.0 International license.

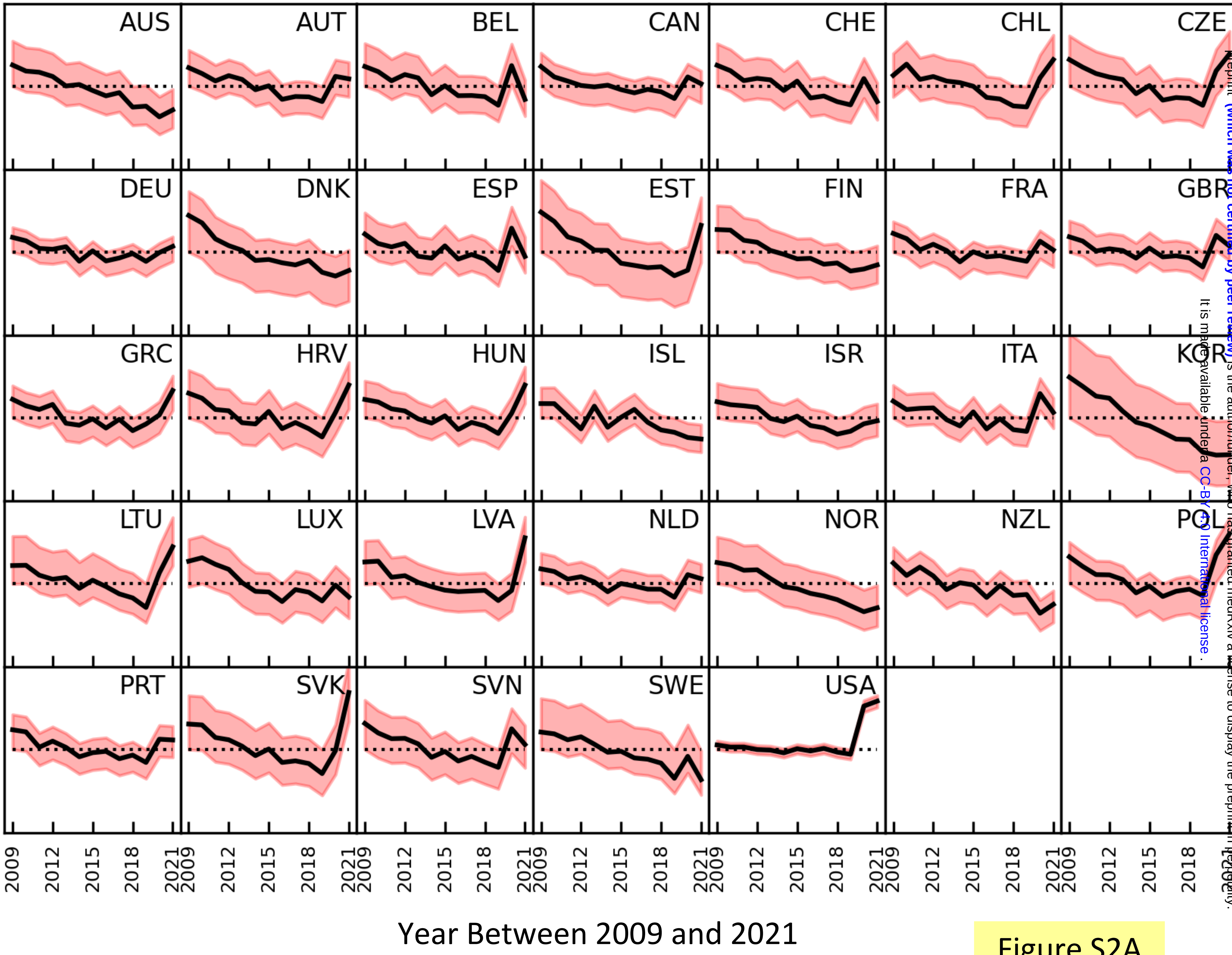

Figure S2A

medRxiv preprint doi: https://doi.org/10.1101/2022.09.21.22280219; this version posted September 23, 2022. The copyright holder for this preprint (which was not certified by peer review) is the author/funder, who has granted medRxiv a license to display the preprint in perpetuity.
It is made available under a CC-BY 4.0 International license .

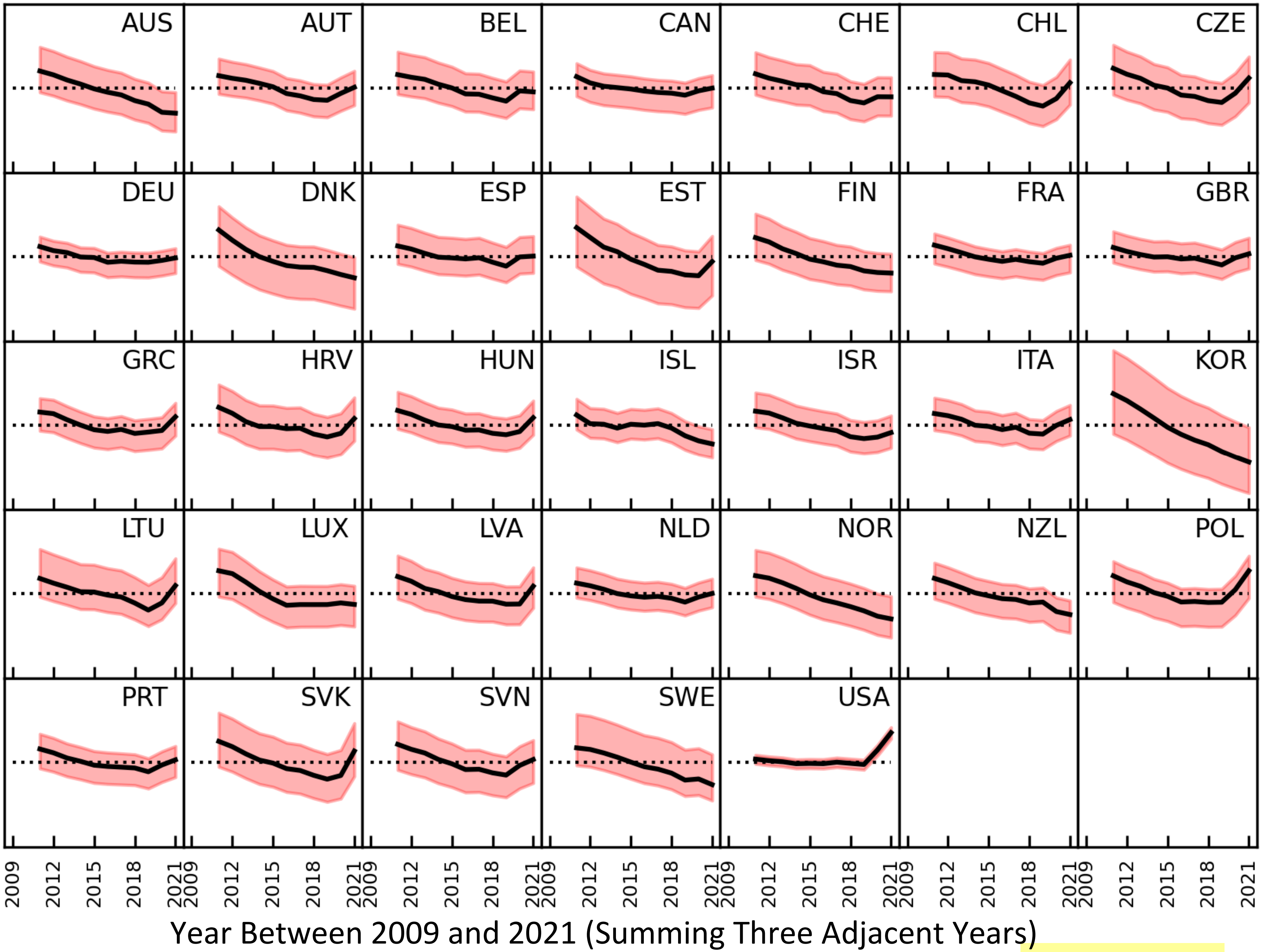

Figure S2B

medRxiv preprint doi: https://doi.org/10.1101/2022.09.21.22280219; this version posted September 23, 2022. The copyright holder for this preprint (which was not certified by peer review) is the author/funder, who has granted medRxiv a license to display the preprint in perpetuity.
It is made available under a CC-BY 4.0 International license .

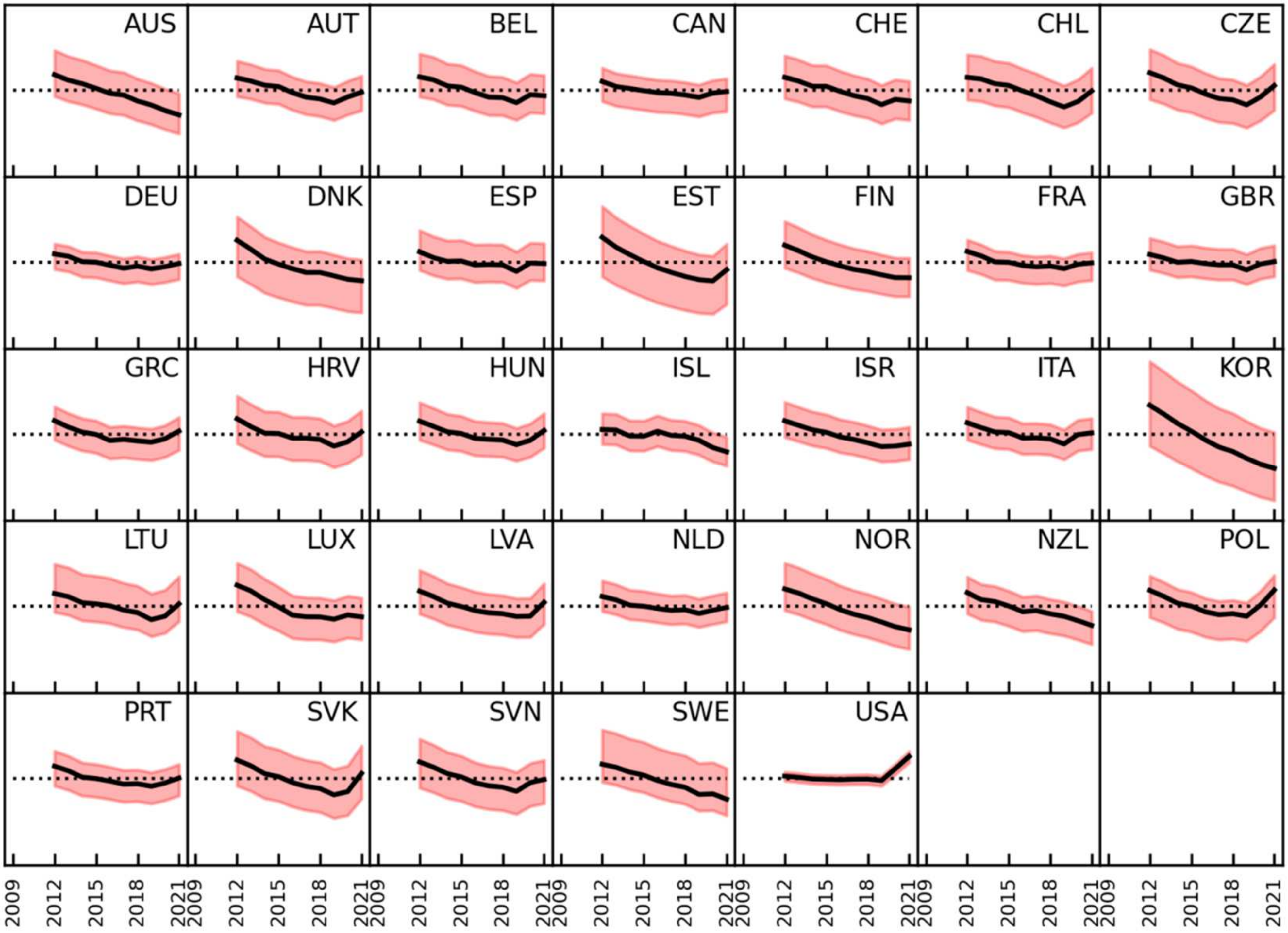

Year Between 2009 and 2021 (Summing Four Adjacent Years)

Figure S2C

medRxiv preprint doi: https://doi.org/10.1101/2022.09.21.22280219; this version posted September 23, 2022. The copyright holder for this preprint (which was not certified by peer review) is the author/funder, who has granted medRxiv a license to display the preprint in perpetuity.
It is made available under a CC-BY 4.0 International license .

Supplementary Links to Data

Location	LOC	Death File Name	Last Modification Date	Population File Name	Last Modification Date	Death Download Link	Population Download Link
All HMD Short Term Mortality Fluctuations		stmf.csv	20-May-2022	stmf.csv	20-May-2022	https://www.mortality.org/File/GetDocument/Public/STMF/Outputs/stmf.csv	
Australia	AUS	Australia_Deaths_1x1.txt	22-Mar-2022	Australia_Population.txt	22-Mar-2022	AUS.Deaths_1x1.txt	AUS.Population.txt
Austria	AUT	Austria_Deaths_1x1.txt	30-Mar-2021	Austria_Population.txt	30-Mar-2021	AUT.Deaths_1x1.txt	AUT.Population.txt
Belgium	BEL	Belgium_Deaths_1x1.txt	25-Sep-2021	Belgium_Population.txt	25-Sep-2021	BEL.Deaths_1x1.txt	BEL.Population.txt
Canada	CAN	Canada_Deaths_1x1.txt	28-Sep-2021	Canada_Population.txt	28-Sep-2021	CAN.Deaths_1x1.txt	CAN.Population.txt
Switzerland	CHE	Switzerland_Deaths_1x1.txt	28-Oct-2021	Switzerland_Population.txt	28-Oct-2021	CHE.Deaths_1x1.txt	CHE.Population.txt
Chile	CHL	Chile_Deaths_1x1.txt	18-Apr-2022	Chile_Population.txt	18-Apr-2022	CHL.Deaths_1x1.txt	CHL.Population.txt
Czechia	CZE	Czechia_Deaths_1x1.txt	23-May-2021	Czechia_Population.txt	23-May-2021	CZE.Deaths_1x1.txt	CZE.Population.txt
Germany	DEU	Germany_Deaths_1x1.txt	17-Dec-2018	Germany_Population.txt	17-Dec-2018	DEUTNP.Deaths_1x1.txt	DEUTNP.Population.txt
Denmark	DNK	Denmark_Deaths_1x1.txt	22-Mar-2022	Denmark_Population.txt	22-Mar-2022	DNK.Deaths_1x1.txt	DNK.Population.txt
Spain	ESP	Spain_Deaths_1x1.txt	23-Feb-2022	Spain_Population.txt	23-Feb-2022	ESP.Deaths_1x1.txt	ESP.Population.txt
Estonia	EST	Estonia_Deaths_1x1.txt	21-Jan-2021	Estonia_Population.txt	21-Jan-2021	EST.Deaths_1x1.txt	EST.Population.txt
Finland	FIN	Finland_Deaths_1x1.txt	02-Aug-2021	Finland_Population.txt	02-Aug-2021	FIN.Deaths_1x1.txt	FIN.Population.txt
France	FRA	France_Deaths_1x1.txt	11-Apr-2022	France_Population.txt	11-Apr-2022	FRATNP.Deaths_1x1.txt	FRATNP.Population.txt
United Kingdom	GBR	UK_Deaths_1x1.txt	11-Jul-2020	UK_Population.txt	11-Jul-2020	GBR_NP.Deaths_1x1.txt	GBR_NP.Population.txt
Greece	GRC	Greece_Deaths_1x1.txt	08-Nov-2021	Greece_Population.txt	08-Nov-2021	GRC.Deaths_1x1.txt	GRC.Population.txt
Croatia	HRV	Croatia_Deaths_1x1.txt	24-Feb-2022	Croatia_Population.txt	24-Feb-2022	HRV.Deaths_1x1.txt	HRV.Population.txt
Hungary	HUN	Hungary_Deaths_1x1.txt	30-Nov-2021	Hungary_Population.txt	30-Nov-2021	HUN.Deaths_1x1.txt	HUN.Population.txt
Iceland	ISL	Iceland_Deaths_1x1.txt	02-Apr-2020	Iceland_Population.txt	02-Apr-2020	ISL.Deaths_1x1.txt	ISL.Population.txt
Israel	ISR	Israel_Deaths_1x1.txt	31-Oct-2018	Israel_Population.txt	31-Oct-2018	ISR.Deaths_1x1.txt	ISR.Population.txt
Italy	ITA	Italy_Deaths_1x1.txt	11-Apr-2022	Italy_Population.txt	11-Apr-2022	ITA.Deaths_1x1.txt	ITA.Population.txt
South Korea	KOR	Republic_of_Korea_Deaths_1x1.txt	15-Nov-2019	Republic_of_Korea_Population.txt	15-Nov-2019	KOR.Deaths_1x1.txt	KOR.Population.txt
Lithuania	LTU	Lithuania_Deaths_1x1.txt	29-Jan-2022	Lithuania_Population.txt	29-Jan-2022	LTU.Deaths_1x1.txt	LTU.Population.txt
Luxembourg	LUX	Luxembourg_Deaths_1x1.txt	21-Jan-2022	Luxembourg_Population.txt	21-Jan-2022	LUX.Deaths_1x1.txt	LUX.Population.txt
Latvia	LVA	Latvia_Deaths_1x1.txt	11-Mar-2021	Latvia_Population.txt	11-Mar-2021	LVA.Deaths_1x1.txt	LVA.Population.txt
Netherlands	NLD	Netherlands_Deaths_1x1.txt	31-Mar-2021	Netherlands_Population.txt	31-Mar-2021	NLD.Deaths_1x1.txt	NLD.Population.txt
Norway	NOR	Norway_Deaths_1x1.txt	15-Apr-2021	Norway_Population.txt	15-Apr-2021	NOR.Deaths_1x1.txt	NOR.Population.txt
New Zealand	NZL	New_Zealand_Deaths_1x1.txt	26-Sep-2017	New_Zealand_Population.txt	26-Sep-2017	NZL_NP.Deaths_1x1.txt	NZL_NP.Population.txt
Poland	POL	Poland_Deaths_1x1.txt	13-Apr-2021	Poland_Population.txt	13-Apr-2021	POL.Deaths_1x1.txt	POL.Population.txt
Portugal	PRT	Portugal_Deaths_1x1.txt	01-Aug-2021	Portugal_Population.txt	01-Aug-2021	PRT.Deaths_1x1.txt	PRT.Population.txt
Slovakia	SVK	Slovakia_Deaths_1x1.txt	29-Oct-2021	Slovakia_Population.txt	29-Oct-2021	SVK.Deaths_1x1.txt	SVK.Population.txt
Slovenia	SVN	Slovenia_Deaths_1x1.txt	01-Nov-2021	Slovenia_Population.txt	01-Nov-2021	SVN.Deaths_1x1.txt	SVN.Population.txt
Sweden	SWE	Sweden_Deaths_1x1.txt	12-May-2022	Sweden_Population.txt	12-May-2022	SWE.Deaths_1x1.txt	SWE.Population.txt
United States	USA	USA_Deaths_1x1.txt	17-Mar-2021	USA_Population.txt	17-Mar-2021	USA.Deaths_1x1.txt	USA.Population.txt

All Deaths_1x1.txt and Population.txt files are downloaded as a zip file at **https://www.mortality.org/File/Download/hmd.v6/zip/all_hmd/hmd_statistics_20220812.zip**, where the Version shown here is "hmd_statistics_20220812" and changes frequently. The relevant files for Deaths and Population are in directories /<Version>/deaths/Deaths_1x1/ and /<Version>/population/Population/ respectively. More generally, the download button for the latest version is marked "All HMD Statistics" and is at the bottom of the page linked by **https://www.mortality.org/Data/ZippedDataFiles**

medRxiv preprint doi: https://doi.org/10.1101/2022.09.21.22280219; this version posted September 23, 2022. The copyright holder for this preprint (which was not certified by peer review) is the author/funder, who has granted medRxiv a license to display the preprint in perpetuity.
It is made available under a CC-BY 4.0 International license .
